# *In situ* oxidative stress in patients with epiretinal membrane

**DOI:** 10.3389/abp.2024.13581

**Published:** 2024-10-18

**Authors:** Tomasz Wilczyński, Jolanta Zalejska-Fiolka, Sabina Sapeta-Wieckowska, Monika Sarnat-Kucharczyk, Wojciech Rokicki

**Affiliations:** ^1^ Department of Ophthalmology, Professor K. Gibinski University Clinical Center, Medical University of Silesia, Katowice, Poland; ^2^ Department of Biochemistry, Faculty of Medical Sciences in Zabrze, Medical University of Silesia, Zabrze, Poland; ^3^ Department of Ophthalmology, Faculty of Medical Sciences in Katowice, Medical University of Silesia, Katowice, Poland

**Keywords:** oxidative stress, epiretinal membrane, retina, total oxidant status, macular hole

## Abstract

**Context:**

Oxidative stress is an important factor for vitreomacular interface disease development in a theoretical model.

**Purpose:**

The aim of the study was to evaluate the correlation between oxidative stress in the human epiretinal membrane (ERM) and retinal morphological changes.

**Material and methods:**

The study included patients scheduled for vitrectomy with epiretinal membrane removal. LogMAR best corrected visual acuity was assessed and optical coherence tomography was performed. Patients were divided into three groups: Type 1 – epiretinal membrane with premacular fibrosis; type 2 – epiretinal membrane with co-existing layer hole; and type 3 – ERM with co-existing full-thickness macular hole. During vitrectomy, epiretinal membranes were collected. Total oxidant status was determined by an automated colorimetric method in homogenates of epiretinal membrane.

**Statistical analysis:**

The Mann-Whitney U test, Kruskal-Wallis test and Spearman linear correlation analysis were used. Statistical significance was set with a level of α = 0.05.

**Results:**

Twenty-one Caucasian women (60%) and 14 men (40%) were included in the study. The average age of participants was 74.7 years (95% CI: 71.13–75.45). The mean best corrected visual acuity LogMAR value in the group was 0.8 (95% CI: 0.9–0.7). The mean ratio of total oxidant status to protein level in the collected samples was 0.161 (95% CI: 0.08–0.23) µmol/mg of protein. No correlation was found between total oxidant status and the degree of morphological retinal changes.

**Conclusion:**

The study found no significant correlation between the level of oxidative stress in epiretinal membrane and retinal morphological changes.

## Introduction

Epiretinal membrane (ERM) is a disease occurring predominantly among patients over 50 years old. It may originate from disorders such as retinal tear, branch or central retinal vein occlusion, and diabetic retinopathy. However, it may also occur without a known reason, and then is termed idiopathic. Previously published data on ERM prevalence in ethnic and age groups vary in the range 2%–29% (Stevenson et al., 2016; Bu et al., 2014; Xiao et al., 2017).

Idiopathic ERM typically consists of a cellular part on the vitreous chamber side and a layer of extracellular matrix (Bu et al., 2014). The exact etiology of ERM is unknown. The cellular layer is mainly composed of retinal glial cells, which have probably migrated to its surface through microdamage of the internal limiting membrane, secondary to posterior vitreous detachment (PVD). Vitreous cortex hyalocytes are the second significant group of cells remaining on the internal limiting membrane (ILM) surface in the course of PVD. Moreover, the presence of macrophages and fibroblasts is found. Myofibroblasts are an important cellular ingredient, originating through the differentiation of glial cells or hyalocytes. They are responsible for formation of a significant part of the ERM extracellular matrix and in further stages for its contraction (Chen and Lee, 2008; Snead et al., 2004; Smiddy et al., 1989).

Another theory of ERM formation is the RPE cells migration through hidden breaks into the inner retina. However, there is currently no direct evidence to support this theory (Wang et al., 2015). Next possible theory suggests that other cell types, such as glial cells, may transform into RPE cells. These GFAP-positive cells could originate from hyalocytes or Müller cells (Bringmann and Wiedemann, 2009; Zhao et al., 2013).

Cellophane maculopathy is the early, asymptomatic stage of idiopathic ERM where the retinal architecture is not considerably affected. In advanced stages the growth and shrinkage of membranes result in retinal fold formation, its swelling, and retinoschisis, and can lead to the formation of holes. The condition is described as macular pucker or premacular fibrosis (Xiao et al., 2017). The coexistence of ERM and lamellar or full-thickness holes may be related to oxidative stress (Chen and Lee, 2008).

Metabolic processes allowing for the correct functioning of cells are largely based on oxidation reactions. They naturally result in the creation of the reactive oxygen species (ROS) which play a crucial role in numerous physiological processes such as the functioning of the immune system, cellular growth, proliferation, differentiation or apoptosis (Wert et al., 2018). An excess of ROS has a highly toxic impact on cells. Thus, its production is balanced predominantly by the activity of anti-oxidation mechanisms. The natural system designed to balance the toxic influence of free oxygen radicals is based on a system of a dozen enzymes. An excess of these reactive oxygen species may be reduced by two anti-oxidation systems, enzymatic and non-enzymatic (Wert et al., 2018; Beyazyıldız et al., 2013; Erel, 2005; Altinisik et al., 2018).

Oxidative stress is an imbalance between reactive oxygen species production and their neutralization capabilities (Altinisik et al., 2018).

Rokicki et al. (2016) reported an important association between oxidative stress and glaucoma. Other studies have described the connection of oxidative stress with retinal diseases such as retinitis pigmentosa, retinal diabetes complications as well as retinal vessel diseases, e.g., central retinal vein occlusion (Erel, 2005; Martínez-Fernández de la Cámara et al., 2013; Guidry et al., 2003).

Retinal photoreceptor cells are a cellular population which is one of the most sensitive to oxidative stress in the body (Guidry et al., 2003). This particular vulnerability results from the fact that the retina is one of the most metabolically active human tissues additionally subjected to constant ultraviolet radiation exposure. As a result it produces particularly large amounts of ROS. Moreover, due to its structure and limited contact with the vascular system, the removal of free radicals is particularly ineffective. This is primarily related to the internal retinal layers where the maximization of tissue transparency takes place at the expense of vascularization. It is associated with the most intensive ROS production (Wert et al., 2018; Guidry et al., 2003). Internal layers of the retina are rich in extracellular free radicals, scavengers of vitreous cortex, and their damage may lead to the development of vitreomacular interface diseases (Kohno et al., 2009).

Total oxidant status (TOS) is one of the methods of describing the oxidative stress value, expressing the total oxidation activity of the tested material (Altinisik et al., 2018).

The objective of our research was to assess *in situ* oxidative stress in human tissues, in the direct vicinity of internal retinal layers. For the purposes of the study, it was hypothesized that the presence of lamellar and full-thickness holes is associated with greater oxidative stress than the presence of ERM itself.

## Material and methods

The ethics committee of the Medical University of Silesia, Katowice (permission number: KNW/0022/KB1/85/III/18/19) approved the study protocol. The study adhered to the tenets of the Declaration of Helsinki for experiments involving human tissue samples. Written informed consent was obtained from all patients after explanation of the nature and possible consequences of the study.

Posterior vitrectomy with removal of ERM and ILM around the macula was performed in 35 eyes from 35 patients. The first collected structure during the surgery was directed for the examination, with its character determined based on interoperative dying of the retina internal limiting membrane at least twice with the MembraneBlue Dual DORC dye as ERM or ERM/ILM complex collected *en bloc*.

The criteria for inclusion in the study group were: age 65–80 years, white race, patient eligibility for ERM and/or ILM removal, informed consent to the procedure, and written consent to participate in the study.

The exclusion criteria were metabolic and inflammatory diseases in medical history, features of active inflammation measured by C-reactive protein (CRP) activity, and obesity above BMI 29.99.

Depending on the pre-surgery retinal morphology the cases were divided into three types. Type 1 – patients with diagnosed ERM presence with premacular fibrosis (PMF), type 2 – patients with ERM with co-existing lamellar hole, type 3 – patients with ERM with co-existing full-thickness macular hole.

For each patient LogMAR best corrected visual acuity (BCVA) was assessed. Optical coherence tomography (Cirrus HD-OCT 5000, Carl Zeiss Meditec, Dublin, CA) was performed to assess the morphology and describe central retina thickness parameters.

Analysis of Total Oxidant Status (TOS) was performed in ERM homogenate and was conducted using a PerkinElmer automated analyzer (PerkinElmer, Waltham, MA, United States) in according to Erel (2005). This method excludes the influence of solution dilution on the TOS value. The assay is based on the oxidation of ferrous ion to ferric ion in the presence of various oxidant species in acidic medium. The change in color of the ferric ion by xylenol orange is measured as a change in absorbance at 560 nm. The automated analyzer Perkin Elmer was calibrated with hydrogen peroxide. The TOS value was expressed in µmol/mg of protein.

Statistical data analysis was conducted using tools available in the Statistica v. 12.0 software. Quantitative variables are presented as the mean and 95% confidence interval. Statistical significance of differences in quantitative variables between two groups was estimated based on the Mann-Whitney U test and in the case of comparison of three groups the Kruskal-Wallis test. Spearman linear correlation analysis was used for the simple analysis of correlation between quantitative variables. The interpretation of results of simple statistical significance tests of differences and correlations was conducted with the adopted significance level α = 0.05.

## Results

The examined group consisted of 21 Caucasian women (60%) and 14 men (40%). The average age of participants in the whole group was 74.7 years (95% CI: 71.13–76.45). There were no statistically significant differences between the groups of men and women. Three groups were distinguished among the patients, based on the preoperative retinal morphology in OCT. Type 1 was represented by 18 people with a mean age of 74.11 (95% CI: 69.06–77.15); type 2 included 11 people, with a mean age of 75 (95% CI: 70.09–79.9); type 3 was represented by six people, with a mean age of 74.66 (95% CI: 66.99–82.34). Age differences among the groups were not statistically significant.

The mean postoperative BCVA LogMAR value in the entire study group was 0.8 (95% CI: 0.9–0.7). For type 1 the mean BCVA was 0.8 (95% CI: 1.0–0.6), for type 2 the mean BCVA was 0.6 (95% CI: 0.9–0.5), for type 3 the mean BCVA was 1.4 (95% CI: 1.7–1.2). A statistically significant difference was found between types 3 and 2 (*p* = 0.001) and between types 3 and 1 (*p* = 0.03).

The average ratio of total TOS to protein level in the collected sample was 0.161 (95% CI: 0.08–0.23) µmol/mg protein. The analysis by group showed no statistically significant differences. The *p*-value for comparisons between the three groups was 0.3. The values of average TOS in relation to the amount of material collected, divided into groups, are presented in Table 1. There was no statistically significant relationship between the age of participants and the tested parameters (*p* > 0.05). The highest TOS value was in group 3 and was 0.28 μmol/mg protein. The mean TOS value in patients with ERM was 0.09 (95% CI: 0.03–0.15). However, the average TOS value in people with ERM/ILM was 0.17 (95% CI: 0.06–0.28). The *p*-value for the correlation between ERM and the ERM/ILM complex was 0.73.

**TABLE 1:** Total oxidant status in groups.

Disease type	Parameter	Mean	95% CI
Type 1	TOS/protein [µmol/mg]	0.12	0.03–0.20
Type 2	TOS/protein [µmol/mg]	0.15	(−0.05) −0.37
Type 3	TOS/protein [µmol/mg]	0.28	0.09–0.47

The average value of central retinal thickness (CRT) in the entire group was 410.8 μm (95% CI: 373.74–447.85). When analyzing correlations with TOS parameters, protein concentration and age, no statistically significant correlations were observed. When divided into groups according to the type of disease, there was a statistically significant difference between the CRT value in patients with type 1 (460.22 μm) and type 3 disease (304.16 μm; *p* = 0.012). Detailed parameters in the groups are presented in Table 2.

**TABLE 2:** Central retinal thickness.

Disease type	Central retinal thickness – CRT (μm)	95% CI
Type 1	460.22	415.20–505.23
Type 2	388.09	448.99–287.00
Type 3	304.16	192.86–415.47

## Discussion

To the best of our knowledge this is the first study to assess the total oxidant status *in vivo* in internal retinal layers and in ERM directly associated with them. It is also the first study to determine the oxidative stress level in eyes with ERM depending on their morphology and degree of central retinal deformation estimated in the OCT examination.

The retinal membranes (pathological structure) and the internal limiting membrane (one of the layers of the retina) are the material closest to the nervous tissue of the eye that can be collected and evaluated *in vivo* in the immediate vicinity of the macula. The collection of the above membranes is part of the surgical procedure and the resulting limitation is a negligible amount of material that is obtained for testing. In our study we decided to assess the correlation between morphological changes of the retina and TOS. Previous studies demonstrated that in patients with ERM the mean central retinal thickness significantly corresponds with visual acuity. This constitutes an objective and repetitive parameter obtained in the OCT examination and is the basis of ERM severity grading systems (Stevenson et al., 2016).

We did not detect a significant relationship between the amount of oxidative stress, expressed by TOS *in situ*, and the thickness of the retina in the place where the material for testing was taken. We also found no statistically significant differences between TOS in the case of the co-occurrence of lamellar or full-thickness holes in the central retina. The determined TOS values do not show any dependence on the severity of morphological changes in the retina.

The myofibroblasts in the ERM cell layer are responsible for its tendency to contract, resulting in the formation of holes in the retina and/or its wrinkle and edema. They are derived from differentiated retinal glial cells and hyalocytes of the posterior hyaloid membrane (Kohno et al., 2009; Abrahan et al., 2009).

Abrahan et al. (2009) observed *in vitro* the effect of oxidative stress on the differentiation and proliferation of retinal microglia and Müller cells. In our *in vivo* model, we examined only one parameter of oxidative stress at the time of an already developed ERM and an advanced disease process known as macular wrinkle or PMF. The small amount of material obtained during the surgical procedure did not allow for the determination of a greater number of markers of oxidative stress with the biochemical methodology applied in this study.

The influence of oxidative stress on the function of the retina *in vivo* in an animal study was demonstrated by Berkowitz (Berkowitz et al., 2019). They found damage to the retinal light response in mice with pharmacologically induced oxidative stress, regressing with administration of antioxidants. Although the reduction of oxidative stress in the animal model led to the improvement of the functional retina, the available human studies did not confirm the effect of orally administered antioxidants on the improvement of retinal morphology parameters in optical coherent tomography (Garcia-Medina et al., 2015).

Available *in vivo* studies on humans refer to single oxidative stress marker assessment in patients with diabetic retinopathy. Collected samples of vitreous body or secondary ERM were compared with idiopathic ERM (Augustin et al., 1995; Lu et al., 2014).

When examining TOS, we did not find any relationship between the severity of changes in OCT and the state of oxidative stress in the retina. The advantage of our study is primarily the attempt to assess the state of oxidative stress *in situ*, which does not always correspond to the systemic state (Lavi et al., 2008; Davel et al., 2012). In our opinion, it gives a more precise picture of changes induced by oxidative stress in such specific oxygen conditions as the vitreoretinal region.

### Study limitations

The main limitation of the study was the lack of measurement of TOS in both the ERM and the peripheral blood. However, due to the strong blood-retina barrier, oxidative stress in the eye may not reflect oxidative stress in the whole body.

The very strong point of this study is the measurement of oxidative stress *in vivo*, i.e., in the eye tissues, precisely in the ERM, and not using *in vitro* method. This pilot study included only 35 patients. A broader study is planned, including a larger group of patients, taking into account TOS parameters in both eye tissues and peripheral blood, and assessing their relationship.

## Conclusion

The degree of epiretinal membrane does not directly depend on the state of oxidative stress.

## Data Availability

The raw data supporting the conclusions of this article will be made available by the authors, without undue reservation.
